# Engineered anti-cancer nanomedicine for synergistic cuproptosis-immunotherapy

**DOI:** 10.1016/j.mtbio.2025.102133

**Published:** 2025-07-29

**Authors:** Jiaxin Li, Xinlong Pang, Zhongwei Xin, Liang Song, Xiangyan Liu, Xinyu Zhang, Zhe Yang

**Affiliations:** a Tumor Research and Therapy Center, Shandong Provincial Hospital, Cheeloo College of Medicine, Shandong University, Shandong, Jinan, 250022, China; b Tumor Research and Therapy Center, Shandong Provincial Hospital Affiliated to Shandong First Medical University, Shandong, Shandong, Jinan, 250022, China; c Department of Thoracic Surgery, Shandong Provincial Hospital, Cheeloo College of Medicine, Shandong University, Shandong, Jinan, 250022, China; d Department of Thoracic Surgery, Shandong Provincial Hospital Affiliated to Shandong First Medical University, Shandong, Shandong, Jinan, 250022, China

**Keywords:** Nanomedicine, Cuproptosis, Immunotherapy, Tumor microenvironment, Cancer treatment

## Abstract

Tumor immunotherapy has seen remarkable progress in recent cancer treatments. However, its broad clinical application is hindered by several challenges, such as the low immunogenicity of "cold tumors," immunosuppressive conditions within the tumor microenvironment, and persistent immune evasion of cancer cells. Therefore, selectively enhancing the immune response during cancer therapy is crucial. Cuproptosis is a newly identified form of regulated cell death that is characterized by the accumulation of intracellular copper ions, leading to lipoylated protein aggregation and subsequent metabolic dysfunction. This process is intricately linked with the tumor microenvironment. By activating targeted mechanisms, cuproptosis can selectively trigger immune responses within tumor regions, thereby addressing the issue of tumor-mediated immune evasion. Cuproptosis holds promise for improving the efficacy of immunotherapy by promoting a more robust anti-tumor immune response within the tumor microenvironment. The combination of cuproptosis-inducing strategies with immunotherapeutic approaches presents a novel and potentially effective direction for future cancer treatment.

## Highlights

• Various cancer cuproptosis-immunotherapy nanomaterials are introduced.
• The mutual interaction between cuproptosis-immunotherapy is highlighted.
• The challenges of cuproptosis -immunotherapy nanomaterials are highlighted.
• Future potential of cuproptosis -immunotherapy nanomaterials is prospected.

## Graphical abstract

Herein, an overview of the research progress in engineered nanomaterials for synergistic cancer cuproptosis-immunotherapy was carried out. The mechanism of those nanomaterials triggering tumor cuproptosis and amplifying antitumor immune response was highlighted. This review can further promote the development of cuproptosis-immunotherapy nanomedicine from basic scientific research to clinical transformation.

**Figure:**
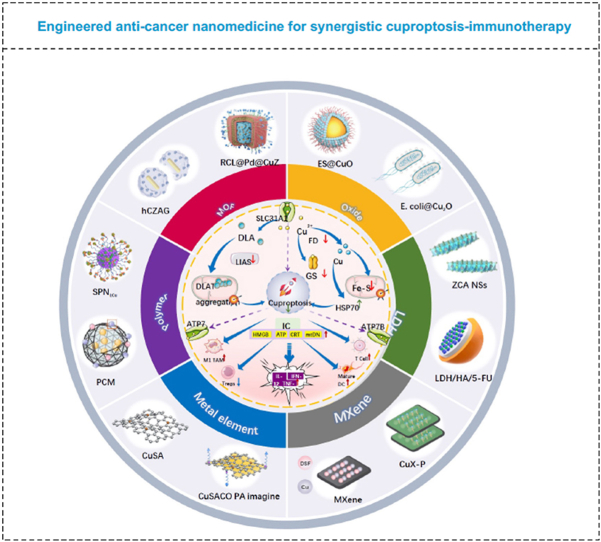

## 1 Introduction

### 1.1 Current status and challenges of cancer immunotherapy

Cancer remains a significant global health challenge, presenting complex obstacles to effective treatment [1]. Cancer immunotherapy represents a groundbreaking approach that harnesses the patient's immune system to specifically target and eradicate malignant cells. This strategy has demonstrated remarkable clinical efficacy across multiple cancer types and effectively addresses key limitations associated with traditional therapies [2]. Immunotherapy offers novel therapeutic options for cancer, providing distinct advantages over conventional strategies such as radiotherapy, chemotherapy, and targeted therapy: (1) It induces a targeted immune response against tumor cells by activating the immune system. For instance, immune checkpoint inhibitors (ICIs) release the inhibitory signals that suppress T-cell activity, thereby enhancing tumor cell recognition and elimination [3,4]. (2) Immunotherapy promotes long-term activation of immune memory, enabling sustained surveillance of residual cancer cells after treatment, which contributes to the prevention of disease recurrence and extends disease-free survival [[5], [6], [7]]. Collectively, these findings underscore the transformative potential of immunotherapy as a cornerstone in modern oncology.

Immunotherapy marks a pivotal advancement toward personalized and effective cancer treatment strategies. However, several major challenges still hinder its clinical application [8]: (1) TME harbors immunosuppressive networks—including regulatory T cells (Tregs), myeloid-derived suppressor cells (MDSCs), and immunosuppressive cytokines such as TGF-β and IL-10—that weaken anti-tumor immune surveillance and facilitate tumor immune escape. (2) Physical barriers, such as the dense fibrotic stroma and components of the extracellular matrix (ECM), restrict effector T lymphocytes' access to tumor cells by inhibiting transendothelial migration and intertumoral infiltration. (3) "Cold tumors," which are defined by low mutational load, impaired antigen presentation, and minimal infiltration of tumor-infiltrating lymphocytes (TILs), exhibit intrinsic resistance to immune checkpoint blockade (ICB). (4) Therapeutic application and dosing optimization are hindered by interpatient variability in treatment response, which stems from genetic, microbiomic, and immune phenotypic differences, as well as the occurrence of immune-related adverse events (irAEs), ranging from mild dermatitis to life-threatening organ toxicities. To overcome immune evasion and fully harness the therapeutic potential of cancer immunotherapy, integrated strategies incorporating immunogenic cell death (ICD) induction, stromal remodeling, and microbiota modulation must be developed.

### 1.2 The concept and related regulatory mechanisms of cuproptosis

Programmed cell death is essential for organismal development, tissue homeostasis, and responses to external stimuli. Unlike necrosis, which occurs due to injury or stress, it is an active, genetically controlled process that ensures timely and regulated cell termination, supporting normal physiology and health. Well-established types include apoptosis, necroptosis, pyroptosis, ferroptosis, and cuproptosis. Cuproptosis, a discovered form described by Tsvetkov et al., involves copper ionophores increasing intracellular copper levels, leading to lipoylated protein aggregation, mitochondrial dysfunction through iron-sulfur cluster protein impairment, and proteotoxic stress. [9]. Compared to other forms of programmed cell death, such as apoptosis, necroptosis, pyroptosis, and ferroptosis, cuproptosis is characterized by unique subcellular morphological changes, including mitochondrial crumpling, cell membrane rupture, endoplasmic reticulum damage, and chromatin fragmentation [10]. Copper acts as a cofactor for enzymes involved in cellular respiration, antioxidant defense, and neurotransmitter synthesis [11]. Under normal conditions, copper homeostasis is maintained by SLC31A1, ATP7A/B, and GSH, which mediate copper uptake, efflux, and intracellular chelation, respectively [12]. Excess copper binds to acylated components of the TCA cycle, leading to aggregation of thiol-acylating proteins and loss of iron-sulfur cluster proteins in respiratory complexes, thereby inducing toxic stress and cell death. Cuproptosis is tightly linked to mitochondrial protein lipoylation, a conserved post-translational modification of lysine residues that is essential for key TCA cycle enzymes such as DLAT. Ferredoxin 1 (FDX1) interacts with lipoyl synthase (LIAS) to regulate lipoylation and catalyze the reduction of Cu^2+^ to Cu^+^. Cu^+^ initiates cuproptosis by inhibiting mitochondrial respiration through promoting protein aggregation and Fe-S cluster degradation, while also enhancing HSP70 expression to amplify proteotoxic stress and drive cell death.

Cuproptosis proceeds through three core mechanisms: the induction of ROS-mediated oxidative stress, mitochondrial dysfunction, and the amplification of inflammatory signaling networks via positive feedback loops. Compared to other forms of cell death, cuproptosis features a distinct biochemical pathway that can bypass certain drug resistance mechanisms, making it a promising strategy for targeting refractory and recurrent cancers [13]. Here, we highlight cuproptosis as a regulatory hub and explore its functional interplay with other types of programmed cell death.

Apoptosis is a common form of programmed cell death (PCD), characterized by cellular shrinkage, nuclear fragmentation, and the formation of apoptotic bodies [14]. Key regulatory steps in apoptosis include the modulation of pro-apoptotic proteins and the activation of caspase cascade reactions. Additionally, mitochondrial damage caused by cuproptosis subtly regulates apoptosis. Altered mitochondrial membrane permeability releases cytochrome *c* (Cytc), which binds with cytoplasmic Apaf-1 to form the apoptosome. This complex activates caspase-9 and subsequently downstream caspase-3, initiating the intrinsic apoptosis pathway [15].

Necroptosis functions as a compensatory cell death mechanism when apoptosis is impaired under inflammatory, oxidative, or ischemic stress. Current evidence indicates that the initiation and regulation of necroptosis primarily involve proteins including TNF-α (tumor necrosis factor-alpha), caspase-8, RIPK1 (receptor-interacting protein kinase 1), RIPK3, and MLKL (mixed lineage kinase domain-like protein). During cuproptosis, copper ions interact with these core necroptotic regulators. Specifically, Cu^+^ activates the RIPK3 pathway, initiating its phosphorylation and downstream MLKL signaling cascade. Moreover, cuproptosis-induced mitochondrial damage leads to the release of damage-associated molecular patterns (DAMPs), such as high mobility group box 1 protein (HMGB1), heat shock proteins, and mitochondrial DNA (mtDNA). These DAMPs propagate inflammatory responses through positive feedback mechanisms, thereby promoting necroptotic cell death [16].

Pyroptosis is a Gasdermin-dependent form of programmed necrotic cell death that leads to inflammatory cell lysis. Activation of NLRP3 promotes Caspase-1-mediated cleavage of GSDMD, resulting in the formation of N-terminal membrane pores [17]. During cuproptosis, excessive reactive oxygen species (ROS) are generated and oxidize specific sites on NLRP3, thereby facilitating inflammasome assembly. Concurrently, mitochondrial dysfunction disrupts the tricarboxylic acid (TCA) cycle, leading to ATP depletion, which further activates NLRP3. Additionally, the release and activation of inflammatory mediators such as DAMPs synergistically enhance NLRP3 activity. Collectively, cuproptosis amplifies NLRP3 activation through multiple converging pathways, culminating in Caspase-1 activation. Activated Caspase-1 cleaves GSDMD, thereby inducing pore formation and executing pyroptosis [18].

Ferroptosis is an iron-dependent form of regulated cell death driven by lipid peroxidation, with the inactivation of glutathione peroxidase 4 (GPX4) serving as a central initiating event [19]. Cuproptosis and ferroptosis are linked through shared pathways involving mitochondrial metabolism, metal ion balance, and oxidative stress. Cuproptosis produces ROS that convert Fe^2+^ into reactive radicals, which catalyze lipid peroxidation via the Fenton reaction. In turn, lipid peroxidation byproducts boost ROS production, creating a positive feedback loop. This cycle continues until lipid peroxides exceed GPX4's capacity, triggering ferroptosis. Additionally, cuproptosis inhibits GPX4 via two mechanisms: (1) Copper binds directly to GPX4, lowering its activity; and (2) Mitochondrial dysfunction reduces ATP, downregulating SLC7A11 and limiting cystine uptake. As a result, GSH synthesis declines, accelerating GPX4 inactivation and inducing ferroptosis [20].

### 1.3 The mutual regulatory effect between cuproptosis and immunotherapy

Copper performs two critical functions in cells: as an essential trace element and as a cofactor for key enzymes involved in physiological processes. Cancer cells reprogram copper metabolism, resulting in elevated labile copper levels within tumors compared to normal tissues. This dysregulation impairs immune cell function in TME and promotes oncogenic signaling. Significantly, excessive copper accumulation leads to the aggregation of lipoylated proteins in the TCA cycle, ultimately inducing cell death. Cuproptosis distinguishes itself from other forms of cell death by circumventing classical effector mechanisms while simultaneously engaging multiple stress response pathways. Targeted delivery of copper via nanocarriers offers a promising therapeutic approach by disrupting intratumoral copper homeostasis, which reverses immunosuppression, stimulates antitumor immune responses, and activates multiple pathways of cell death. This coordinated effect transforms immunologically quiescent 'cold' tumors into immunologically active 'hot' tumors, enhancing the potential for effective cancer immunotherapy. Currently, various nanomedicines are under investigation for their capacity to induce cuproptosis in cancer (Table 1). Future research will focus on refining nanomedicine design, enhancing therapeutic efficacy, and facilitating clinical translation.

**Table 1:** Representative summary in engineered nanomaterials for cancer therapy by regulating cuproptosis pathways.

Cuproptosis pathways	Biomaterials	Cuproptosis reagents	Cuproptosis mechanism	Cancer types	Therapy	Ref.
Copper metabolism → Oxidative stress	CuT/ET HNP	CuET	Cu^2+^↓→GSH↓→Capase3/7↑	4T1 cells	NCT	[21]
	CuMoO_4_	Cu^+^	Cu^2+^↓→GSH↓→Capase3/7↑	MCF-7, 4T1 cells	PTT	[22]
	ZCProP	Cu^2+^	Cu^2+^↓→GSH↓→Capase3/7↑	4T1 cells	CDT	[23]
	AuTPyP-Cu	Cu^2+^	Cu^2+^↓→GSH↓→Capase3/7↑	HeLa cells	SDT	[24]
	CS/NPs	Cu^2+^	Cu^2+^↓→GSH↓→Capase3/7↑	MCF-7, 4T1 cells	CDT	[25]
	GOx@[Cu(tz)]	Cu^+^, Cu^2+^	Cu^2+^↓→GSH↓→Capase3/7↑	MCF-7, 5637 cells	PDT	[26]
	Cu_2_O@Mn_3_Cu_3_O_8_(CMCO)	Cu_2_O	Cu^2+^↓→GSH↓→Capase3/7↑	L929, CT26 cells	PTT	[27]
	CuSAzyme	Cu-O_2_/Cu-O_4_	Cu^2+^↓→GSH↓→Capase3/7↑	CT26 cells	NCT	[28]
	AuPt@Cu-PDA	Cu^2+^	Cu^2+^↓→GSH↓→Capase3/7↑	4T1 cells	NCT/PTT	[29]
	Cu–Fe_3_O_4_	Cu^2+^	Cu^2+^↓→GSH↓→Capase3/7↑	HUVEC cells	CDT	[30]
	NCTD Gel	Cu^2+^	Cu^2+^↓→GSH↓→Capase3/7↑	HepG2 cells	CDT	[31]
Copper metabolism → Autophagy	DCM@GDY-CuMOF@DOX	Cu^+^	ROS↑ →Bax↑, Capase3/9↑, Bcl-2↓→Capase12↑	DU145 cells	CDT	[32]
	HFn-Cu-REGO	Cu^2+^	ROS↑ →Bax↑, Capase3/9↑, Bcl-2↓→Capase12↑	GBM cells	CDT	[33]
	CSTD-Cu(II) @DSF	Cu^2+^	ROS↑ →Bax↑, Capase3/9↑, Bcl-2↓→Capase12↑	MCF-7 cells	CDT	[34]
Copper metabolism → Apoptosis	Au@MSN-Cu/PEG/DSF	Cu^2+^	ROS↑ →Bax↑, Capase3/9↑, Bcl-2↓→Capase12↑	4T1 cells	PTT	[35]
	HA-CuH-PVP	Cu^+^	ROS↑ →Bax↑, Capase3/9↑, Bcl-2↓→Capase12↑	4T1, A549, B16, CT26 cells	NCT	[36]
	T-HCN@CuMS	Cu^+^, Cu^2+^	ROS↑ →Bax↑, Capase3/9↑, Bcl-2↓→Capase12↑	143B cells	PDT	[37]
	CuO	Cu^2+^	ROS↑ →Bax↑, Capase3/9↑, Bcl-2↓→Capase12↑	HTR8, Svneo cells	PTT	[38]
	HD/BER/GOx/Cu	Cu^2+^	ROS↑ →Bax↑, Capase3/9↑, Bcl-2↓→Capase12↑	4T1 cells	CDT	[39]
Copper metabolism → Immunotoxicity	CAT-ecSNA-Cu	Cu^+^	Cu↑→p-EGFR↑→PD-L1↑	CT26 cells	NCT	[40]
	ES-Cu/Galactose-Alginate (CSG)	Cu^2+^	ES↑→Cu↓→PD-L1↓	CT26 cells	PDT	[41]

*Note.* NCT:Nano-catalytic Therapy. SDT:Sonodynamic Therapy. CDT:Chemodynamic Therapy. PDT: Photodynamic Therapy. PTT: Photothermal Therapy.

In 2023, Jin et al. proposed that cuproptosis could mediate systemic immune responses, reshape the immunosuppressive TME to prevent immune evasion. To validate this concept, they constructed a nanoreactor designated CCJD-FA, thereby advancing research into cuproptosis-mediated immunotherapy [42]. Subsequently in 2025, Zhou et al. developed CBPNs@HA nanoparticles. These particles leverage the cuproptosis cascade to significantly amplify oxidative stress, effectively inducing ICD in tumor cells and activating systemic anti-tumor immunity. Collectively, these studies provide novel perspectives for cancer immunotherapy [43]. Cuproptosis combined with immunotherapy demonstrates significant therapeutic potential [14]. Emerging evidence reveals a critical interplay between cuproptosis and immune responses, which is essential for advancing cancer therapy. Cuproptosis regulates antitumor immunity through three primary mechanisms: (1) cytokine modulation, (2) depletion of immunosuppressive cells, and (3) activation of immune effector cells. The oxidative stress induced by cuproptosis triggers the release of DAMPs, such as ATP and HMGB1, which stimulate the production of inflammatory cytokines including IL-6, TNF-α, IFN-γ, and IL-1β.

These cytokines promote dendritic cell maturation, enhance antigen presentation, activate cytotoxic T lymphocytes (CTLs), and support memory T cell differentiation. Concurrently, they drive M1 macrophage polarization while inhibiting M2 phenotypes, transforming immunologically 'cold' tumors into 'hot' tumors and reversing immune suppression. Additionally, Proteotoxic stress from cuproptosis activates the mtDNA-cGAS-STING pathway, boosting both innate and adaptive immunity to block tumor immune escape. Preclinical studies show that combining copper-based nanomaterials with αPD-L1 significantly outperforms monotherapy, suppressing up to 100 % of subcutaneous tumors and even controlling metastases. A clinical trial for melanoma is currently investigating the combination of PD-1 inhibitors with the cuproptosis inducer Elesclomol. Accumulating research also suggests that inflammatory signals can reprogram cellular metabolism to increase susceptibility to copper accumulation, thereby promoting cuproptosis. This bidirectional interaction underscores how integrating cuproptosis with immunotherapy overcomes the limitations of single-modality treatments.

### 1.4 Engineered nanomedicine for synergistic cuproptosis-immunotherapy

Advances in materials science have expanded the use of nanotechnology across multiple fields, including optics [44], energy [45], environment, agriculture [46], food [47], and tumor diagnosis and treatment [48]. In combined tumor therapy, nanotechnology shows great promise due to its precise tumor targeting, efficient drug accumulation, strong response to TME, and synergistic multimodal treatment capabilities. As a result, nanomaterial-based combination therapy has become a promising strategy in cancer treatment [[49], [50]].

**Fig. 1:**
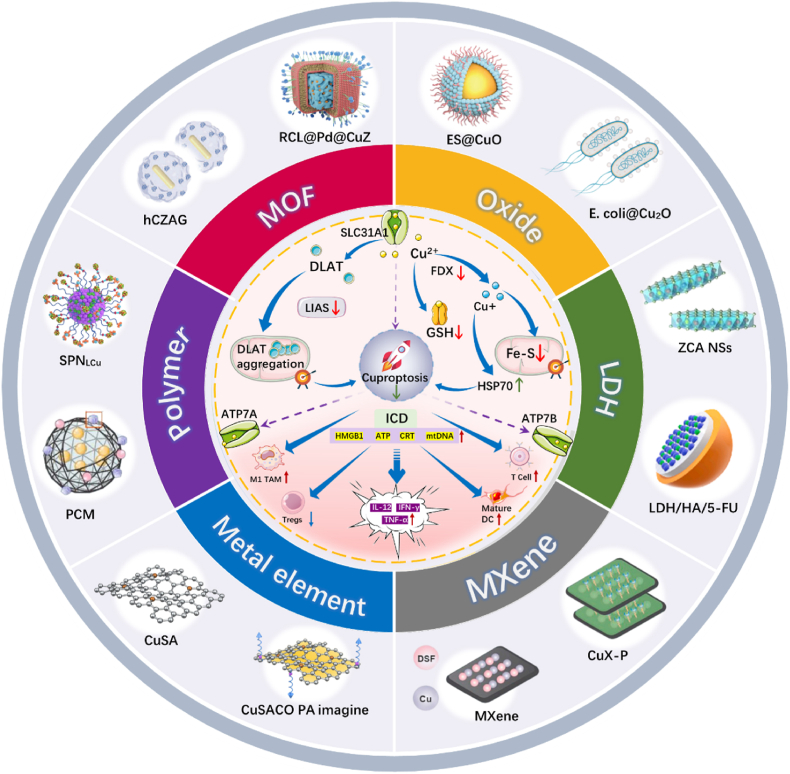
An overview of engineered anti-cancer nanomedicine for synergistic cuproptosis-immunotherapy.

However, limited attention has been paid to the optimization of nanomaterials for synergistic cuproptosis-immunotherapy. This review presents a systematic classification and analysis of engineered nanomedicines developed for this combined therapeutic strategy (Table 2), including metal-organic frameworks (MOFs), metal oxides, metallic elements, polymeric nanomaterials, layered double hydroxides (LDHs), and MXene materials (Fig. 1). It offers a comprehensive summary of their roles and underlying mechanisms in tumor therapy, with the aim of serving as a valuable reference and stimulating further innovation. Although nanomaterials hold great promise for cuproptosis-based immunotherapy, they also face substantial challenges in design and application. By synthesizing recent progress in cuproptosis-immune-associated nanomaterials, this review elucidates the regulatory interplay between cuproptosis and immune responses, thereby guiding the rational design of nanomaterials tailored for effective combination therapy.

**Table 2:** Summary of nanomedicine for cuproptosis/immunotherapy.

Types	Biomaterials	Component	Immune pathways	Immune mechanism	Toxicity Detection Methods	Preparation Methods	Therapeutic Efficiency	Degradation Method	Degradation Time	Cu in tumor	Cu outside tumor	Ref.
Cu-MOF	OMP	Cu^+^	ICD, PD-L1 upregulation	Active CD8^+^ T cells↑, PD-L1↑	Cytotoxicity, blood and histological analysis	Liquid exfoliation and sonication treatment	75 %	GSH, pH	24 h	1.5 μmol/L	0.26 μmol/L	[51]
	BCMD	Cu^2+^	Reverse TIME, ICD	DCs↑, CD8^+^ T cells↑, IFN-γ↑	Cytotoxicity, blood and histological analysis	Liquid-solid-solution method	67 %	pH	24 h	6.3 μg/g	0.3 μg/g	[32]
	RCL@Pd@CuZ	Cu^2+^	Reverse TIME	CD4^+^ T cells↑, CD8^+^ T cells↑	Blood and histological analysis	Biomineralization-inspired Synthesis	Almost 100 %	GSH	unknown	unknown	unknown	[52]
	GOX	Cu^2+^	Reverse TIME	DCs↑, CD4^+^ T cells↑, CD8^+^ T cells↑	Cytotoxicity, blood and histological analysis	Mineralization Synthesis	Almost 100 %	GSH	unknown	unknown	unknown	[53]
Copper oxide nanomaterials	E. Coli@Cu_2_O	Cu^2+^	ICD	Mature DCs↑, CTLs↑	Blood analysis	Framework Exchange strategy	Almost 100 %	GSH	unknown	unknown	unknown	[54]
	Es@CuO	Cu^2+^	Reverse TIME, ICI	IL-6↑、TNF-*α*↑, IFN-γ↑	Cytotoxicity, blood and histological analysis	Hydrothermal Method	67 %	pH	unknown	unknown	unknown	[55]
Cu (0)	CuSACO	Cu^2+^	ICD	DCs↑, active T cells↑, M1 phenotype↑	Blood and histological analysis	In-situ Bombardment Embedding Technology	Almost 100 %	GSH	24 h	unknown	unknown	[56]
Polymer nanoparticles	CQG NPs	Cu^2+^	Reverse TIME	Mature DCs↑, active T cells↑, M1 phenotype↑	Cytotoxicity, blood and histological analysis	Surfactant-assisted wet chemistry strategy	Almost 100 %	GSH	unknown	unknown	unknown	[57]
	PCM	Cu^2+^	Activating STING pathway	Mature DCs↑、CD8+ T cells↑, IFN-γ↑	Blood analysis	Liquid exfoliation and sonication treatment	Almost 100 %	Proteotoxic	24 h	160 ng	2 ng	[58]
	SPNLCu	Cu^2+^	Reverse TIME/ICD	Efficacy of ICD↑, CD3^+^ T cells、CD8+ T cells	Cytotoxicity, blood and histological analysis	Amide Coupling Reaction	Almost 100 %	ROS,pH	24 h	unknown	unknown	[59]
LDH	LDH/HA/5-FU	Cu^2+^, 5-FU	Reverse TIME	M1 phenotype↑,Active CD4^+^ T cells↑, CD8^+^ T cells↑	Cytotoxicity, blood and histological analysis	Hydrothermal	70 %	pH	unknown	unknown	unknown	[60]
	ZCA NS	Cu^2+^	Reverse TIME/ICD	Mature DCs↑	Cytotoxicity and histological analysis	Coprecipitation method	Almost 100 %	GSH	12 h	unknown	unknown	[61]
	LDH/HA/DOX	Cu^2+^	Reverse TIME	Active CD4^+^ T cells↑, CD8^+^ T cells↑, M1 phenotype↑	Cytotoxicity, blood and histological analysis	Chemical precipitation method and Solvothermal	67 %	GSH	24 h	unknown	unknown	[62]
MXene	CuX-P	Cu^2+^	ICD, PD-L1 downregulation	PD-L1↓	Blood and histological analysis	Selective etching method and layered Method	75 %	Tumor Endocytosis	unknown	unknown	unknown	[63]
	Cu_2_O/Ti_3_C_2_T_X_	Cu^+^	Reverse TIME	ICD↑,DC cells↑	Cytotoxicity, blood and histological analysis	Acid etching method and sonication treatment	Almost 100 %	pH	24 h	Cu^2+^,Cu^+^	unknown	[64]
Complexes	Cu-DBCO/CL	Cu-DBCO	ICD/PD-L1 downregulation	Mature CD8^+^ T cells↑	Cytotoxicity, blood and histological analysis	High-temperature organic-solution phase systhesis	Almost 100 %	ROS	48 h	unknown	unknown	[65]
	NP@ESCu	Cu	ICD/PD-L1 downregulation	PD-1 inhibition	Cytotoxicity, blood and histological analysis	Liquid exfoliation and sonication treatment	Almost 100 %	ROS	unknown	copper increased by 4.2-fold	unknown	[66]

## 2 Diverse engineered nanomedicine for synergistic cuproptosis-immunotherapy

### 2.1 MOF

MOFs are coordination polymers composed of metal ions and organic ligands [67]. Due to their high porosity, large specific surface area, excellent drug-loading capacity, and biodegradability, MOFs have been widely applied in drug delivery, bioimaging, and tissue engineering [68]. Their synthesis typically involves hydrothermal/solvothermal methods, microemulsion techniques, microwave-assisted approaches, or ultrasound-assisted synthesis [69]. Cu-MOF releases Cu^2+^ ions that enhance oxidative stress through the Fenton reaction, leading to ROS generation and cuproptosis by promoting DLAT aggregation. This cuproptosis further enhances immunotherapy efficacy by modulating the tumor immune microenvironment, making Cu-MOF a promising candidate for cancer treatment.

**Fig. 2:**
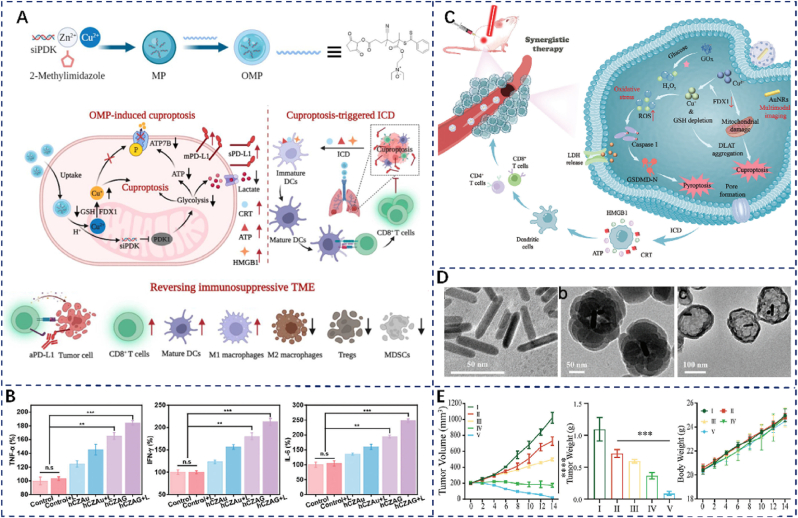
(A) Schematic representation of the OMP for synergistic cuproptosis and immunotherapy. (B) TNF-α, IFN-α, and IL-6 levels in tumor tissues of 4T1 tumor-bearing mice under different treatment regimens. (C) Schematic illustration of hCZAG enabling synergistic cuproptosis, pyroptosis, and immunotherapy. (D) TEM of hCZAG. Images showing a) AuNRs, b) CZAu, and c) hCZAG are presented. Reproduced with permission [54] Copyright 2024, Wiley. (E) In vivo Evaluation of RCL@Pd@CuZ + Anti-PD-1 Synergy: Treatment groups: (I) Radiotherapy (RT), (II) RT + anti-PD-1, (III) RCL@Pd@CuZ + anti-PD-1, (IV) RCL@Pd@CuZ + RT, (V) RCL@Pd@CuZ + RT + anti-PD-1. Reproduced with permission [53] Copyright 2024, Wiley.

Cuproptosis can enhance immunotherapy efficacy by reversing the inhibitory TME. In 2023, In 2023, Yan et al. developed an inhalable Cu-MOF loaded with siPDK, termed OMP (Fig. 2A) [51]. Compared to intravenous injection, inhalation delivery resulted in superior pulmonary accumulation and retention. OMP induces cuproptosis through Cu^+^ release in acidic, GSH-rich conditions and simultaneously delivers siPDK to inhibit glycolysis and suppress ATP7B expression, thereby elevating intracellular Cu^2+^ levels. Western blot analysis revealed significantly reduced expression of ACO-2, Lip-DLAT, DLAT, and FDX1 in the OMP-treated group. Moreover, OMP enhances dendritic cell maturation, cytokine secretion, and CD8^+^ T-cell activation via ICD (Fig. 2B). Following 24 h of treatment, B16F10 cells exhibited increased membrane-bound and secreted mPD-L1 levels. The combination of OMP with αPD-L1 demonstrated the most potent anti-metastatic activity. Overall, OMP represents a promising strategy for lung metastasis treatment by integrating cuproptosis with immunotherapy.

Glioblastoma (GBM) is the most common and deadliest primary malignant brain tumor. The blood-brain barrier limits drug penetration into the tumor. Additionally, GBM secretes high levels of laminin and fibronectin, forming a dense stromal network that hinders both therapeutic agent delivery and immune cell infiltration. As a result, GBM is classified as a stroma-rich "cold" tumor. This presents a key challenge: how to overcome resistance to cuproptosis-immunotherapy strategies in such stroma-rich environments. In 2023, Huang et al. reported a novel nanosystem named BCMD for cuproptosis-immunotherapy. The system employs dodecyl β-D-maltoside to encapsulate MOF-199 loaded with BSO and CAT, thereby enhancing nasal absorption [70]. Nasal administration bypasses the blood-brain barrier and significantly improves bioavailability. Within the acidic tumor microenvironment (TME), BCMD releases Cu^2+^ to induce cuproptosis while co-delivering BSO and CAT, which enhance therapeutic sensitivity by reducing GSH levels and increasing O_2_ availability. Experimental data demonstrated that BCMD effectively decreased GSH, elevated O_2_ levels, and induced Fe-S cluster protein depletion along with DLAT aggregation. Treated GL261 cells exhibited increased release of HMGB1 and calreticulin (CRT), accompanied by enhanced infiltration of mature dendritic cells (DCs), CD8^+^ T cells, and elevated interferon-gamma (IFN-γ) production. IFN-γ further promoted cuproptosis by suppressing GSH synthesis. Combination therapy with BCMD and αPD-L1 effectively reversed immunosuppression and significantly prolonged survival. This study advances the development of MOF-based cuproptosis-immunotherapy and provides key insights into nanoparticle design for targeted brain tumor therapy.

In 2024, Wang et al. developed a hollow-core yolk-shell nanoplatform (hCZAG) by encapsulating ZIF-8 with AuNRs and using Cu^2+^ and Zn^2+^ as active nodes (Fig. 2C and D) [53]. This system induces cuproptosis, which in turn triggers cell pyroptosis and ICD. Glucose oxidase consumes glucose to produce H_2_O_2_, while Cu^2+^ induces cuproptosis through the Fenton reaction, depletes GSH, and promotes ROS generation. Excess ROS activates Caspase-1, leading to pyroptosis. Compared to controls, the hCZAG + Light group showed increased DLAT aggregation, CRT and HMGB1 exposure, more mature dendritic cells (CD80/CD86), and higher CD4^+^ and CD8^+^ T cell infiltration. It also elevated TNF-α, IFN-α, and IL-6 levels, indicating strong immune activation. Moreover, AuNRs enabled hCZAG to perform photothermal and photoacoustic imaging (PTI/PAI), supporting image-guided tumor therapy. In summary, this work introduces a new strategy combining cuproptosis-induced pyroptosis with immunotherapy, offering insights into improving pyroptosis-based cancer treatments.

Li et al. developed a novel radiosensitizer (RCL@Pd@CuZ) by integrating Cu-MOF encapsulated Pdzyme with phospholipids, RGD-DSPE-PEG, cholesterol, and capsaicin to augment ICD induced by radiation therapy (Fig. 2E) [52]. The nanosystem enhances radiotherapy sensitivity through mitochondrial respiration inhibition, ROS and oxygen generation promotion, and improved X-ray absorption. Compared with conventional radiotherapy, the RCL@Pd@CuZ + RT group exhibited significantly increased CRT and HMGB1 release, as well as enhanced infiltration of CD4^+^ and CD8^+^ T cells, indicating robust ICD induction. Under acidic tumor microenvironment (TME) conditions, released Cu^2+^ induces copper-dependent cell death by binding to TCA cycle components, while also triggering iron-dependent cell death and apoptosis via Fenton reaction activation and GSH depletion. Western blot analysis confirmed downregulation of LIAS, FDX1, and GPX4, along with an elevated Bax/Bcl-2 ratio, supporting the activation of multiple cell death pathways. When combined with anti-PD-1 antibody, the treatment led to a significant reduction in tumor volume and weight, with no notable change in mouse body weight. These findings demonstrate that the platform effectively enhances radiation-induced ICD through copper-dependent mechanisms, offering a promising strategy to overcome radioresistance and advance immune-oncology therapies.

MOFs have been widely applied across various fields such as biosensors, wound healing, antimicrobial agents, drug delivery, and cancer therapy, owing to their distinctive structural and functional advantages. [[71], [72], [73], [74], [75], [76]]. The MOF-based materials discussed in this section for cuproptosis-immunotherapy (including OMP, BCMD, hCZAG, and RCL@Pd@CuZ) predominantly utilize Cu-MOFs. This preference stems from Cu-MOFs' ability to undergo responsive degradation in the weakly acidic TME releasing Cu^2+^ ions to induce cuproptosis by elevating copper levels within the TME. Notably, OMP and BCMD are administered via inhalation and intranasal delivery, respectively. This direct mucosal administration route accelerates drug absorption, bypasses the blood-brain barrier (BBB) for direct brain targeting, and reduces systemic side effects, offering distinct advantages over conventional intravenous administration. Furthermore, hCZAG concurrently activates both cuproptosis and pyroptosis—two novel cell death pathways—enabling it to combat refractory and recurrent malignant tumors by circumventing specific drug resistance mechanisms. RCL@Pd@CuZ incorporates palladium (Pd) to synergize with radiotherapy, enhancing immune cell infiltration and demonstrating significant potential for promoting cold-to-hot tumor transformation. Consequently, integrating multiple therapeutic modalities constitutes the underlying design principle for engineering nanomaterials in cuproptosis-immunotherapy. However, as a relatively new research area, MOFs face significant challenges in design, production, and clinical translation. Cu-MOFs that induce cuproptosis show promise for tumor therapy. Yet, their effectiveness and application are limited by instability under high temperatures and corrosive conditions, potential toxicity in the body, and difficulties in scaling up production [68]. Overcoming these limitations and advancing the clinical application of Cu-MOFs are key priorities for future research. Therefore, optimizing nanostructure design to improve safety and targeting is essential for developing Cu-MOFs in cuproptosis-immunotherapy.

### 2.2 Metal oxide

CuO nanoparticles (NPs) have attracted significant attention in antibacterial, antioxidant, and anticancer research due to their potent toxicity, high drug-loading capacity, and excellent stability [77,78]. These NPs induce cuproptosis, stimulate ROS production, and facilitate stable and efficient intracellular accumulation of Cu^2+^ ions, particularly in tumor therapy settings [79]. CuO NP-based nanosystems play a crucial role in cuproptosis-immunotherapy by offering a straightforward yet highly effective approach.

**Fig. 3:**
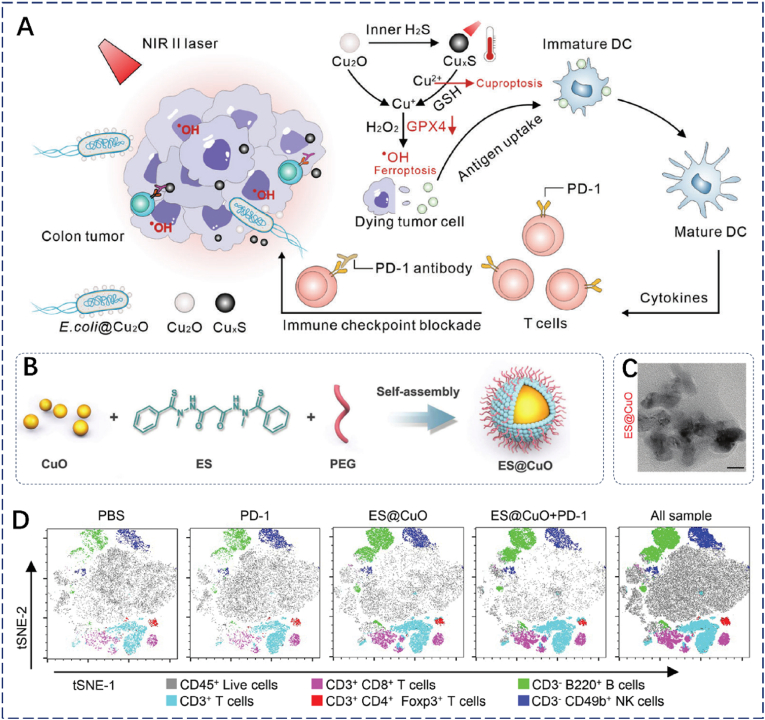
(A) E. coli@Cu_2_O for synergistic immunotherapy of ferroptosis and cuproptosis. Reproduced with permission [55]. Copyright 2023, Wiley. (B) Schematic of ES@CuO NPs preparation. (C) Representative TEM images of ES@CuO NPs. (D) T-SNE analysis of B16 tumor tissues under different treatments. Reproduced with permission [56]. Copyright 2024, Wiley.

In the TME of colorectal cancer, In 2023, Ruan et al. developed a nanosystem, E. coli@Cu_2_O, which uses Escherichia coli as a carrier to deliver Cu_2_O specifically to the tumor site, thereby enhancing photothermal therapy (PTT), inducing cuproptosis and ferroptosis, and modulating the immune microenvironment [54] (Fig. 3A). Initially, Initially, Cu_2_O demonstrated superior near-infrared absorbance compared to CuO. Furthermore, Cu_2_O effectively reacted with H_2_S to form Cu_x_S, which exhibited excellent photothermal conversion efficiency, leading to GSH depletion and ROS generation. I Immunohistochemical analysis revealed significantly reduced GPX4 expression, DLAT protein aggregation, and CRT exposure in the E. coli@Cu_2_O group under 1064 nm laser irradiation, indicating induction of both ferroptosis and cuproptosis as well as activation of ICD. Flow cytometry results showed that this treatment induced the highest levels of DCs and CTLs, demonstrating that PTT enhances immune responses mediated by ferroptosis and cuproptosis. In a bilateral MC38 mouse tumor model, no tumor growth was observed in either primary or secondary tumors following treatment with E. coli@Cu_2_O, 1064 nm laser irradiation, and PD-L1 antibody, suggesting effective prevention of tumor recurrence and metastasis. Overall, this study presents a novel strategy to overcome challenges in immune checkpoint blockade therapy and provides new insights into copper-based oxides for cuproptosis-driven immunotherapy.

In 2024, Lu et al. developed a nanoplatform, Es@CuO, to enhance anti-tumor immunity through cuproptosis induction. The platform encapsulates CuO with Elesclomol (ES), a copper ion carrier, and is coated with PEG (Fig. 3B and C) [55]. Under acidic conditions, ES@CuO releases Cu^2+^ ions that accumulate in cancer cells, disrupt membrane integrity, and trigger cuproptosis, thereby promoting ICD and improving the efficacy of immune checkpoint inhibitors. Experimental data revealed downregulation of FDX1, aggregation of DLAT, and elevated release of HMGB1, lactate dehydrogenase, and ATP. When combined with anti-PD-1 therapy, ES@CuO significantly increased tumor-infiltrating lymphocytes and upregulated key cytokines (IL-6, TNF-α, IFN-γ), confirming its ability to reprogram the immunosuppressive tumor microenvironment via cuproptosis. This strategy effectively enhances PD-1-based immunotherapy (Fig. 3D). Overall, the study presents a promising approach to overcoming immune checkpoint inhibitor resistance in melanoma and underscores the therapeutic potential of copper oxides in cuproptosis-immune synergistic treatment.

The metal oxide-based materials discussed in this section for cuproptosis-immunotherapy (specifically E. coli@Cu_2_O and ES@CuO) primarily utilize copper oxides. E. coli@Cu_2_O is synthesized by hybridizing Escherichia coli (E. coli) with Cu_2_O NPs. This hybridization endows the material with the colonizing ability of E. coli, thereby promoting drug absorption. Furthermore, Cu_2_O exhibits unique advantages in fields such as PTT due to its excellent near-infrared (NIR) light absorbance and its characteristic reaction with H_2_S to form copper sulfides (Cu_x_S). By enhancing the synergistic antitumor effect of ferroptosis and cuproptosis via PTT, and simultaneously reversing TME immunosuppression, this material holds significant promise for future clinical translation. Additionally, combining ES@CuO with programmed death-1 (PD-1) blockade therapy significantly enhances the antitumor efficacy against murine melanoma. This experimental result substantially advances the clinical translation of combination therapies integrating cuproptosis-inducing nanomaterials with immune checkpoint inhibitors, offering profound clinical implications. Although Cu-oxides show good stability, the potential toxicity of Cu^2+^ remains a critical concern. Factors such as nanoparticle size, shape, surface charge, dosage, and concentration significantly influence the toxicity of CuO/Cu_2_O nanoparticles. While Cu^2+^ can induce cuproptosis and generate ROS to kill tumor cells, it also causes oxidative stress that harms normal cells. Therefore, designing more rational nanosystems for precise Cu^2+^ release and targeted accumulation at tumor sites is essential to address these toxicity challenges.

### 2.3 Copper single-atom materials

Copper-based nanomaterials are mainly synthesized using methods such as chemical treatment, electrochemical synthesis, photochemical techniques, sonochemistry, and heat treatment [80]. Copper NPs are widely applied in optics, electronics, and biomedical fields due to their high abundance, low cost, and ease of preparation. In tumor therapy, elemental copper demonstrates strong tumor enrichment and cytotoxic effects, and has the potential to synergistically improve the overall efficacy of immunotherapy.

**Fig. 4:**
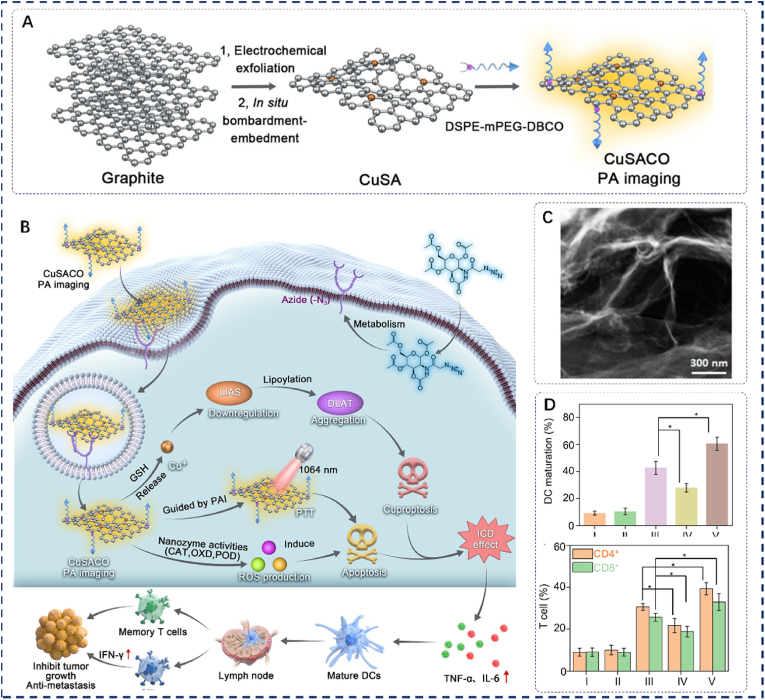
(A) Synthesis of CuSA. (B) Schematic of CuSACO-mediated combination therapy triggering cuproptosis and ICD. (C) Representative low-magnification dark-field TEM image of CuSA. (D) Frequencies of mDCs in dLNs, CD4^+^ T cells, and CD8^+^ T cells across treatment groups:(I) PBS, (II) PBS + light, (III) CuSACO + Ac_4_ManNAz, (IV) CuSACO + Ac_4_ManNAz + NEM, (V) CuSACO + Ac_4_ManNAz + light. Reproduced with permission [57]. Copyright 2024, Wiley.

In 2024, Wu et al. synthesized a novel planar Cu-SAzyme configuration immobilized on monolayer graphene using an in-situ bombardment-embedding technique [56]. Dibenzo cyclooctyne (DBCO) was used to functionalize the structure, resulting in the formation of CuSACO (Fig. 4A). In response to the acidic pH and elevated glutathione (GSH) levels within the tumor microenvironment (TME), CuSACO released Cu^2+^ ions and induced cuproptosis. Western blot analysis confirmed that CuSACO triggered DLAT aggregation and significantly reduced LIAS expression (Fig. 4B and C). Moreover, treatment with Ac4ManNAz enhanced tumor cell uptake of CuSACO. In 4T1 cells treated with CuSACO + Ac4ManNAz, extracellular ATP levels increased, nuclear HMGB1 decreased, and surface CRT expression was elevated, indicating that CuSACO induced immunogenic cell death (ICD) through the cuproptosis pathway (Fig. 4D). Additionally, CuSACO's multi-enzyme activity produced ROS like hydroxyl radicals (·OH), and ^1^O_2_ molecules, which increased the levels of oxidative stress while reducing hypoxia in the TME. This approach further enhanced cuproptosis and immunotherapy effectiveness. CuSACO's strong photothermal properties boosted cuproptosis, ICD, and catalytic activity. In the experiment, it was discovered that the CuSACO + Ac_4_ManNAz + light group successfully induced DC maturation, T cell infiltration, the change from naive to memory T cells, and the conversion of M2-like macrophages into M1-like phenotypes. These findings indicate that PTT, cuproptosis, and nano-catalytic therapy significantly improve the tumor immune microenvironment. This multi-modal immunotherapy shows great potential in suppressing tumor growth, recurrence, and metastasis. Photoacoustic imaging further improves treatment accuracy and efficacy. In conclusion, CuSACO demonstrates excellent potential for multi-modal cancer therapy and provides valuable insights into SAzyme-based cuproptosis-immune combination strategies.

Copper single-atom materials are characterized by straightforward synthesis and excellent photothermal properties. Incorporating various active enzymes into these copper-based atom materials enables the utilization of multiple mechanisms to enhance tumor treatment efficacy. This approach synergistically combines PTT, cuproptosis, and immunotherapy to suppress tumor growth, metastasis, and recurrence. With advantages including exceptional dispersion, high catalytic activity, efficient photothermal conversion, and favorable biocompatibility, copper single-atom materials demonstrate substantial potential for applications in nanomedicine. The CuSACO discussed in this chapter possesses both simple synthesis and efficient photothermal conversion. It can responsively degrade within the TMEto release therapeutic agents. This dual action simultaneously triggers cuproptosis in tumor cells while inducing ICD. Furthermore, CuSACO exhibits multi-enzyme mimetic activity, leveraging diverse mechanisms to enhance anti-tumor efficacy. This facilitates the coordinated application of PTT, cuproptosis, and immunotherapy to synergistically suppress tumor growth, metastasis, and recurrence. Compared to copper-based oxides, elemental copper exhibits advantages in terms of reaction activity and targeting. However, it may be accompanied by increased toxicity and stability concerns. In numerous instances, elemental copper is susceptible to oxidation, leading to the formation of more stable cupric oxide. Consequently, maintaining its stability poses a highly challenging direction for future design and application of Cu NPs.

### 2.4 Polymer nanomaterials

Polymer nanomaterials are composed of a polymer matrix and embedded nanocomponents. They are commonly prepared using methods like electrospinning, template polymerization, solution polymerization, and emulsion polymerization. These materials serve as effective carriers for bioactive agents—such as proteins, nucleic acids, and small-molecule drugs—by protecting them from degradation in the body and preserving their structural integrity before reaching the target. By incorporating copper ions into the matrix, sustained release can be achieved through controlled degradation. The gradual release of low-dose copper ions enhances anti-tumor immunity by promoting dendritic cell maturation and reducing MDSC infiltration, leading to improved therapeutic outcomes. Moreover, copper ions induce tumor cell death through specific mechanisms, including disruption of mitochondrial respiratory chain complexes and accumulation of acetylated proteins, which trigger programmed cell death when concentrations reach toxic levels.

Functional material systems composed of a polymer matrix and additional nanocomponents are commonly referred to as polymer nanomaterials. Their preparation involves conventional techniques such as electrospinning, template polymerization, solution polymerization, and emulsion polymerization. Due to their unique carrier properties, these materials can effectively encapsulate bioactive agents—including proteins, nucleic acids, and small-molecule drugs—resist chemical and enzymatic degradation in vivo, and maintain drug integrity prior to targeted delivery. Incorporating copper ions into the polymer matrix enables sustained release through controlled nanomaterial degradation. The slow release of low-dose copper ions modulates the tumor immune microenvironment in two key ways: first, by promoting dendritic cell maturation and regulating MDSCs infiltration, thereby enhancing anti-tumor immunity and improving therapeutic outcomes. Second, through copper-specific mechanisms such as targeting mitochondrial respiratory chain complexes and inducing aggregation of acetylated proteins, a critical local copper ion concentration can trigger a cascade of programmed cell death in cancer cells.

**Fig. 5:**
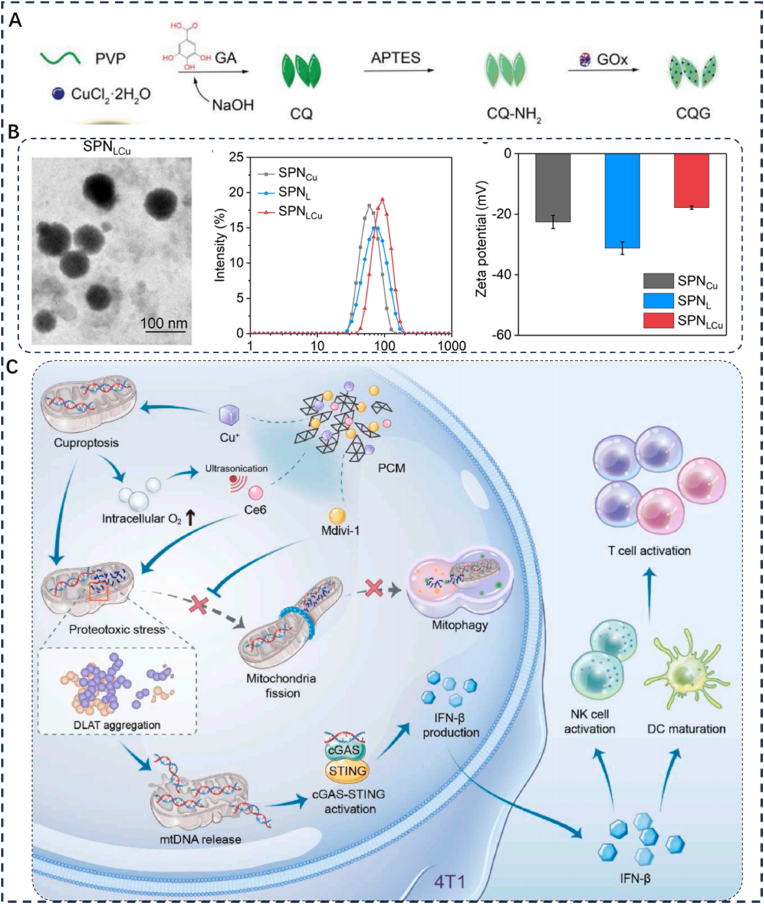
(A) Schematic illustrating the synthesis of CQG NPs. Reproduced with permission [58]. Copyright 2023, Wiley. (B) Characterization of SPNCu, SPNL, and SPNLCu NPs: TEM image, zeta potential, and UV–vis absorption spectra. Reproduced with permission [60]. Copyright 2024, Wiley. (C) Schematic illustration: PCM nanoinducers fabrication and activation of proteotoxic stress-mediated mtDNA–cGAS–STING signaling for potent antitumor immunotherapy. Reproduced with permission [59]. Copyright 2024, Elsevier.

Research has shown that a subset of tumor cells can enter a dormant state during tumor proliferation and expansion. These dormant cells are difficult for the immune system to detect in a timely manner, allowing them to persist and develop significant resistance to therapeutic agents. Consequently, stimulating immune activity and improving the tumor microenvironment are considered key therapeutic strategies for managing recurrent tumors. In 2023, Qiao et al. developed copper quinone cascade nanozyme-glucose oxidase (CQG NPs) with multiple peroxidase activities [57] (Fig. 5A and B). After accumulating at the tumor site, CQG NPs deplete GSH and produce oxygen, which inhibits HIF-1α expression and reduces NQO1 and NRF2 levels, disrupting the antioxidant system in tumor cells. At the same time, these nanoparticles increase ROS production, activating the NLRP3 inflammasome and triggering Caspase-1-mediated cleavage of gasdermin D (GSDMD). This leads to membrane pore formation and release of pro-inflammatory cytokines. As CQG NPs degrade, they release copper ions that promote cuproptosis within tumor cells. The released cytokines shift tumor-associated macrophages from an M2 immunosuppressive state to an M1 immunostimulatory phenotype. They also induce dendritic cell (DC) maturation and enhance T cell infiltration into the tumor microenvironment. This reprogramming of the immunosuppressive TME ultimately triggers strong anti-tumor immune responses in vivo.

Cuproptosis occurs when excessive copper ions accumulate inside cells. Sulfureted proteins in the tricarboxylic acid (TCA) cycle bind to these ions, leading to abnormal protein oligomerization and reduced levels of Fe-S cluster proteins. These changes increase organelle membrane permeability, trigger protein toxicity stress, and result in the release of mitochondrial DNA (mtDNA) into the cytoplasm. Once there, mtDNA activates the cGAS-STING pathway, which enhances the maturation and activation of antigen-presenting cells. In 2024, Yu et al. reduced Cu^2+^ to monovalent Cu^+^, forming a stable Cu-phenol network termed PC-Cu. They further encapsulated the mitotic inhibitor Mdivi-1 into a PEG-polyphenol-chlorin e6 (Ce6) system to construct an amphiphilic PCM nanocarrier [58] (Fig. 5C). Once internalized by tumor cells, the PCM nanocarrier underwent controlled degradation and delivered monovalent copper ions into the intracellular environment. These copper ions, in combination with Ce6, induced mitochondrial-specific protein toxicity, leading to dysfunction characterized by reduced oxygen consumption and increased oxygen retention. Moreover, Mdivi-1 inhibited the ubiquitin-proteasome system, impairing the clearance of toxic proteins and thereby promoting mtDNA release into the cytoplasm. This activation of the mtDNA-cGAS-STING pathway subsequently triggered both innate and adaptive anti-tumor immune responses.

The stroma can comprise over 80 % of the total volume in pancreatic tumors. This disproportionately large matrix forms a dense physical barrier that limits the penetration of drugs into pancreatic tumor tissue. Concurrently, the aberrantly hyperactivated TGF-β/SMAD signaling pathway within pancreatic tumors continuously drives stromal hyperplasia and promotes an immunosuppressive microenvironment. Consequently, a promising drug delivery strategy involves overcoming the stromal barrier while preserving its integrity, releasing therapeutic agents intratumorally, remodeling the stroma, and activating the immune system. Targeting pancreatic cancer—a stroma-rich "cold" tumor—requires such an integrated approach. In 2024, Yu et al. constructed a semiconductor polymer nanoparticle reactor, SPNLCu (Fig. 5A), by conjugating lactate oxidase (LOx) to the surface of semiconductor polymer nanoparticles (SPNs) via ROS-cleavable linkers and chelating Cu^2+^ into the structure [59]. Upon irradiation, the semiconductor polymers generate singlet oxygen (^1^O_2_), which cleaves the ROS-cleavable linkers, releasing LOx and depleting extracellular lactate while producing H_2_O_2_. At the same time, Cu^2+^ is released from the nanoparticles and reduced to Cu^+^ within TME. The resulting Cu ^+^ reacts with H_2_O_2_ to form ·OH radicals, further breaking ROS-cleavable linkers and promoting additional LOx release, thereby alleviating lactate-induced immunosuppression. Moreover, a substantial amount of Cu^+^ is released into the TME, triggering copper-dependent cell death in tumor cells. Animal model studies have demonstrated that SPNLCu enhances anti-tumor immune responses, suppresses subcutaneous pancreatic tumor growth, and effectively inhibits tumor progression and metastasis in situ in both Panc02 and KPC pancreatic cancer models.

The polymer nanomaterials discussed in this section primarily center on copper-based materials for cuproptosis-immunotherapy applications. Among them, CQG NPs cleverly activate the NRF2-NQO1 signaling pathway to disrupt the tumor's antioxidant defense mechanisms and trigger pyroptosis via NLRP3 protein activation. However, the precise engineering of nanomaterials to interact specifically with target proteins for remodeling the tumor immune microenvironment presents significant challenges. Successfully addressing these challenges is crucial for translating material research into clinical practice. Additionally, PCM activates the canonical cGAS-STING immune pathway to stimulate the anti-tumor immune system. Consequently, designing nanomaterials that directly interact with immune pathways has become a major research focus with considerable clinical potential. The most innovative material discussed is SPNLCu, which utilizes ultrasound triggering for the cascade release of synergistic drugs. This system overcomes multiple biological barriers within tumor tissue and adapts to dynamic pathological changes within tumors to achieve progressively enhanced therapeutic efficacy. Its combined application with cuproptosis can effectively activate the body's immune system, providing a promising therapeutic strategy against stroma-rich "cold" tumors. Despite the significant advantages of polymeric materials in nanomedicine, their surface physicochemical properties can activate the human immune system and potentially induce immune dysregulation. Moreover, although medical-grade polymers undergo toxicity screening to eliminate hazardous additives, residual monomers or catalytic subunits may still cause in vivo toxicity and tissue damage. Therefore, the development of polymer materials for targeted cuproptosis-immunotherapy that simultaneously fulfill biocompatibility, controlled degradation, functional stability, and safety requirements has become a key research priority.

### 2.5 LDH

LDHs exhibit excellent stability, material safety, and recyclability. By constructing LDH layer structures using copper ion sheets, negatively charged anions, and solvent molecules, these materials enable targeted delivery of anti-tumor drugs, thereby reducing toxicity in surrounding organs [81]. Upon degradation, LDH releases copper ions that accumulate in TME, inducing copper-mediated cell death and modulating the tumor immune microenvironment. These results highlight the potential of LDH-based systems for advanced anti-tumor therapy.

**Fig. 6:**
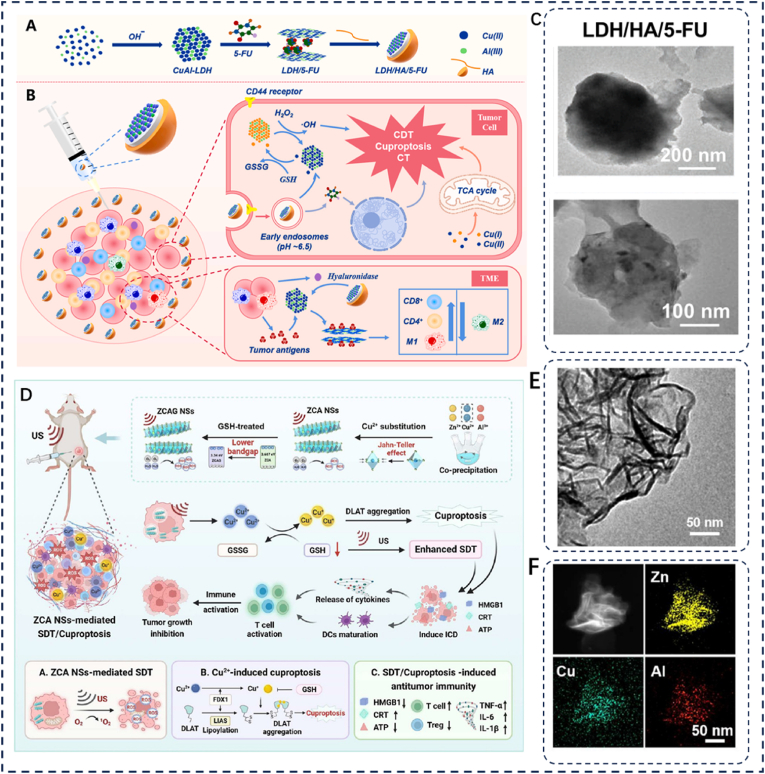
(A–B) Fabrication mechanism of LDH/HA/5-FU NPs: (A) Synthesis process; (B) Anticancer therapeutic action. (C) TEM characterization of LDH/HA/5-FU nanosheets. Reproduced with permission [61]. Copyright 2023, Elsevier. (D) Schematic mechanism of ZCA nanosheets synergizing SDT and cuproptosis for anticancer therapy. (E) TEM characterization of as-synthesized ZCA nanosheets. (F) Elemental mapping (Zn, Al, Cu) of ZCA nanosheets via EDS spectroscopy. Reproduced with permission [63]. Copyright 2024, American Chemical Society.

5-fluorouracil (5-FU), a first-line anti-tumor drug, is commonly employed for chemotherapy in various malignant tumors. However, its non-specificity towards tumor cells and short plasma half-life significantly restricts its clinical utility. In 2024, Xia et al. developed an organic-inorganic hybrid drug delivery system named LDH/HA/5-FU (Fig. 6A–C) [60], which incorporates 5-FU into the interlayer space of CuAl-LDH while adsorbing hyaluronic acid (HA) on the nanoparticle surface to achieve controlled drug release. The resulting LDH/HA/5-FU nanosheets demonstrate excellent biocompatibility and enable targeted therapy through pH-responsive drug release. Additionally, the release of monovalent copper ions enhances copper-induced tumor cell death. Studies have shown that LDH/HA/5-FU can act as an immune modulator, reshaping the tumor microenvironment (TME) and increasing immune cell infiltration. The synergistic integration of chemodynamic therapy (CDT), chemotherapy, and immune activation by LDH/HA/5-FU nanosheets highlights their promising potential for the treatment of solid tumors.

In 2024, Tang et al. developed copper-substituted ZnAl ternary layered double hydroxide nanosheets (ZCA NSs) as a dual-function sonosensitive drug and copper nanocarrier for synergistic anti-tumor therapy by integrating sonodynamic therapy (SDT) with copper-induced cell death (Fig. 6D–F) [61]. These nanosheets enhance oxidative stress by depleting endogenous GSH, thereby improving TME and boosting SDT efficacy. Concurrently, in vivo degradation of ZCA NSs releases a large amount of divalent copper ions, further promoting intracellular copper-induced cell death. Experimental results show that the SDT/cuproptosis combination effectively induces ICD and stimulates dendritic cell (DC) maturation, resulting in a stronger systemic anti-tumor immune response. In CT26 colon cancer and 4T1 breast cancer animal models, this combined therapy completely suppressed lung and liver metastasis of malignant tumors [82].

Tumor cell metabolism generates substantial amounts of lactic acid, creating an acidic TME characterized by chronic immunosuppression. This acidified TME renders tumor cells resistant to conventional chemotherapy. In 2025, Shi et al. developed an innovative LDH-based drug delivery platform (LDH/HA/DOX) for the selective targeting of tumors, promoting DOX release and inducing multiple synergistic anti-tumor effects [62]. This nanocarrier incorporates dopants of Cu^2+^ and Mn^3+^. Mn^3+^ functions as an immunomodulator, activating the cGAS-STING signaling pathway to stimulate an immune response and augment immune cell infiltration within the TME. Conversely, Cu^2+^ downregulates GSH levels, thereby amplifying oxidative stress while simultaneously disrupting copper homeostasis to induce cuproptosis. The combination of DOX with the CuMn-LDH core synergistically targets enhanced ROS generation within the TME, promoting tumor cell apoptosis and overcoming chemoresistance. Both in vitro and in vivo experiments confirmed that LDH/HA/DOX exhibits potent anti-tumor activity and excellent biocompatibility. It precisely targets tumor tissues, remodels the TME, and promotes anti-tumor immunity, achieving synergistic enhancement of CDT, chemotherapy, and immunotherapy [83].

LDH occupies a prominent position in the field of nanomedicine due to its unique layered structure and tunable properties. The LDH-based materials discussed in this section for cuproptosis-immunotherapy (including LDH/HA/5-FU, ZCA NSs, and LDH/HA/DOX) primarily involve copper-loaded LDHs. Among them, LDH/HA/5-FU utilizes hyaluronic acid (HA) adsorbed on its surface to achieve targeted delivery via the HA-CD44 axis [84]. It synergistically induces apoptosis and cuproptosis for anti-tumor therapy while simultaneously improving the infiltration levels of TAMs and T cells in the peritumoral area, demonstrating significant potential for treating solid tumors [85]. Additionally, Cu^2+^ in ZnAl NSs can induce a strong Jahn-Teller effect, ensuring the overall stability of the material. It synergizes SDT and cuproptosis to enhance the anti-tumor effect and stimulate a systemic anti-tumor immune response, completely inhibiting lung and liver metastasis in vitro experiments. LDH/HA/DOX employs Mn^3+^ as an immune function regulator to increase the infiltration of TAMs and T cells. Through the precisely controlled release of the chemotherapeutic drug doxorubicin (DOX), it reduces systemic toxicity and enhances anti-tumor efficacy. While LDHs offer numerous advantages in anti-tumor therapy, their degradation relies on an acidic environment, such as the TME [62]. However, achieving tumor eradication effects in peritumoral tissues remains challenging due to difficulties in precisely controlling their degradation rate and drug release dosage. LDH itself has low immunogenicity, necessitating the addition of immunomodulators to promote intratumoral immune infiltration and ameliorate the immunosuppressive state of "cold" tumors. Therefore, enhancing the release design of LDH and improving its immunogenicity is crucial for the future development of LDH-based materials [86].

### 2.6 MXene

MXenes are a class of two-dimensional transition metal carbides and nitrides with high redox activity, excellent photothermal conversion efficiency, and favorable hydrophilicity [87]. By incorporating copper into the MXene structure as the transition metal component, the material exhibits superior dispersibility in biological fluids. Due to its strong redox properties, it can effectively penetrate TME and be internalized by tumor cells. When combined with PTT, the MXene degrades within tumor cells, disrupts the tumor's antioxidant defense system, and releases a large amount of copper ions, thereby inducing copper-dependent cell death. This process contributes to enhanced immune suppression within the TME.

**Fig. 7:**
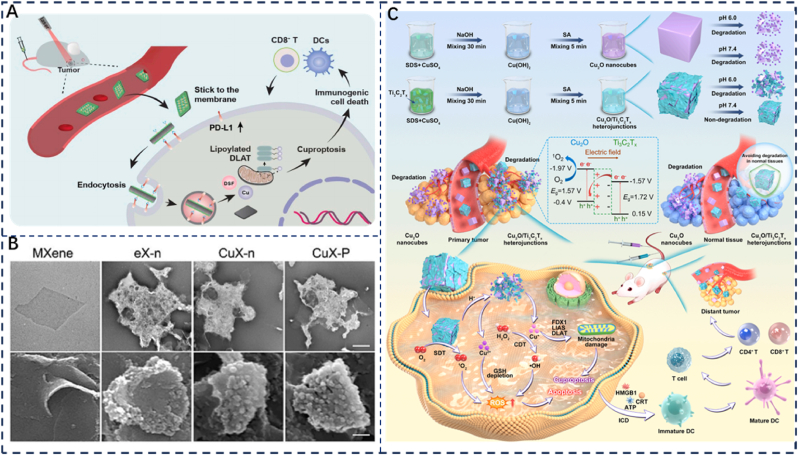
(A) Molecular mechanisms of copper-based compound CuX-P against triple-negative breast cancer. (B) TEM images and SEM images of MXene, eX-n, CuX-n and CuX–P. Reproduced with permission [65]. Copyright 2023, Elsevier. (C) Synthetic strategy and therapeutic mechanism of Cu_2_O/Ti_3_C_2_T_x_ heterojunctions for synergistic sonodynamic-chemodynamic therapy activating cancer immunotherapy. Reproduced with permission [64]. Copyright 2024, Elsevier.

Disulfiram (DSF) is widely used in the treatment of alcoholism and has been shown to chelate divalent copper ions, forming DSF/Cu^2+^ complexes that induce copper-dependent cell death in tumor cells and promote immunogenic cell death (ICD). However, recent studies have revealed that DSF/Cu^2+^ can upregulate PD-L1 expression in tumor cells, facilitating immune evasion. In 2023, Liu et al. developed a CuX-P system by integrating MXene nanosheets loaded with DSF/Cu^2+^ into T-cell membranes overexpressing PD-1 (Fig. 7A and B) [63]. MXene exhibits remarkable photothermal conversion efficiency, electron transparency, and enzyme-triggered degradability, enabling CuX-P to adhere to the surface of tumor cells and specifically recognize/bind with PD-L1, thereby enhancing the internalization of both CuX-P and PD-L1 by tumor cells (Fig. 7C). Accompanied by the internalization of DSF/Cu^2+^ in the cytoplasm, the expression of PD-L1 can be upregulated. Given the existence of CuX-P in TME, PD-L1 on the tumor surface can rebind to CuX-P again. The PD-L1 on the tumor surface is constantly consumed, while CuX-P is continuously enriched in the tumor. The cuproptosis of tumor cells is enhanced, thereby forming a positive feedback loop. Experimental results have confirmed that combining the CuX-P system with laser irradiation effectively induces tumor cell cuproptosis and activates anti-tumor immunity, thereby suppressing tumor recurrence. As a tumor-targeted bionic platform, CuX-P synergistically integrates photothermal therapy (PTT), immunotherapy, and cuproptosis to eliminate cancer cells, demonstrating broad therapeutic potential.

Cuproptosis demonstrates potential in enhancing the efficacy of traditional anticancer therapies and eliciting robust adaptive immune responses. However, the non-tumor-specific release of Cu^2+^ ions can trigger cuproptosis and cause irreversible damage to normal tissues [88]. To better address this challenge, Cao et al. developed a novel nanomaterial, Cu_2_O/Ti_3_C_2_T_x_. They engineered a Z-scheme heterostructure by coating MXene (Ti_3_C_2_T_x_) onto Cu_2_O nanocubes [64]. This strategy not only enhanced the sonodynamic and chemodynamic activities of the Cu_2_O nanocubes but also prevented their degradation under normal physiological conditions. Upon reaching the tumor site, the tumor-specific release of Cu ions from Cu_2_O/Ti_3_C_2_T_x_ achieved cascading amplification of ROS generation, driven by both Cu^+^-mediated Fenton-like reactions and Cu^2+^-facilitated depletion of GSH. Concurrently, it triggered cuproptosis via Cu^+^-induced DLAT oligomerization and mitochondrial dysfunction [89]. More importantly, the high levels of ROS combined with efficient cuproptosis significantly reversed the immunosuppressive TME. This ultimately induced ICD, thereby promoting a potent systemic immune response. This cascade effect resulted in the eradication of the primary tumor and suppression of distant tumors. Collectively, this study provides a promising perspective for potential cancer therapies based on tumor-specific cuproptosis by precisely regulating the degradation behavior of copper-based nanomaterials [90].

MXene demonstrates great potential in nanomedicine owing to its excellent biocompatibility, controllable and safe metabolic profile, and high drug-loading capacity. The MXenes discussed in this section for cuproptosis-immunotherapy, namely CuX-P and Cu_2_O/Ti_3_C_2_T_x_, are primarily Cu-based MXene materials. Among them, CuX-P is endocytosed by adhering to the surface of tumor cells, continuously depleting membrane PD-L1 levels to activate immune cell recognition. It simultaneously combats tumor cells through the synergistic action of PTT and cuproptosis. The development of this bioinspired system enables direct modulation of intracellular PD-L1 in tumor cells to promote ICD, demonstrating considerable significance for the clinical translation of nanomedicine. Additionally, Cu_2_O/Ti_3_C_2_T_x_, with its unique design, not only enhances the structural stability of the material but also strengthens its responsiveness to SDT. This enables precise tumor-targeted therapy through the synergistic integration of multiple regulatory mechanisms. However, a major challenge in MXene fabrication is sheet agglomeration during synthesis, which undermines material uniformity and restricts its scalability for industrial production. Addressing these manufacturing limitations is crucial for achieving large-scale production and advancing the clinical translation of MXene-based therapeutic strategies [91].

## 3 Clinical challenges and translational barriers

Compared to conventional drugs, nanomedicines have much smaller particle sizes and larger surface areas, which pose significant challenges in manufacturing and quality control. Process parameters and scale can greatly affect their safety, quality, and efficacy. Therefore, achieving continuous production and ensuring batch-to-batch consistency are essential.Like other therapeutics, nanomedicines must meet strict regulatory standards for efficacy and toxicity before clinical use. Their manufacturing processes must also comply with current good manufacturing practices (cGMP) [92]. To support the safe development of new nanotherapeutics, regulatory frameworks must be strengthened to ensure robust risk-benefit assessments. In response to the need for standardized guidance, the U.S. Food and Drug Administration (FDA) has issued three draft guidelines covering chemistry, manufacturing, and controls (CMC), preclinical pharmacokinetics, and safety evaluation. The FDA also recommends a risk-based approach for evaluating nanomedicine candidates [93]. Key considerations include full characterization of material properties, performance, in vivo pharmacokinetics, and consistent product quality during manufacturing. An emerging method inspired by systems biology uses omics technologies to collect high-throughput data on how biological systems respond to nanoparticles. This supports systems nanotoxicology and helps develop predictive risk assessment models. Overall, comprehensive evaluation of nano pharmaceuticals should focus on product stability and well-designed clinical trials [94]. Manufacturers using nanomaterials must ensure compliance with safety regulations. Early consultation with regulatory agencies is strongly recommended to address questions about regulatory status, safety, and efficacy. Although the FDA has proposed initial testing methods and standards, more research is needed to fully understand nanoparticle structure-function relationships. As a result, clear characterization standards remain under development, and ongoing advances in the field continue to present new regulatory challenges [95].

Nanomedicine manufacturing parameters must be precisely and individually tailored to improve clinical translation potential [91]. Particle size significantly affects biodistribution: smaller nanomaterials are rapidly cleared through the glomerular endothelium, whereas larger particles are often opsonized and removed by complement proteins before reaching their target [95]. In terms of surface modification, hyaluronic acid (HA) and polyethylene glycol (PEG)-coated copper-based nanomaterials show some tumor-targeting ability [96]. However, HA and PEG can themselves activate immune cells and the complement system, potentially causing hemocompatibility issues such as platelet aggregation [97]. Surface charge also plays a key role: positively charged nanomaterials enhance cellular uptake but may provoke inflammatory responses. For example, layered double hydroxides (LDHs), due to their cationic nature and strong adsorption capacity, tend to accumulate in organs like the liver and spleen [98], which can lead to chronic inflammation, oxidative stress, and fibrosis over time. Biodegradability is another critical factor: nanomaterials for biomedical use must be biologically and mechanically compatible with human tissues [99]. Importantly, their degradation products should not trigger inflammation and must be metabolically eliminable. Compared to non-degradable materials that persist for years, biodegradable nanomaterials offer much greater promise for clinical application [100]. Therefore, precise control over manufacturing parameters—including size, surface chemistry, charge, and biodegradability—is essential for advancing nanomedicine development and achieving successful clinical translation [101].

## 4 Conclusion, discussion, and fresh perspectives

Tumor immunotherapy faces major challenges, including low immunogenicity, poor delivery efficiency, and off-target side effects. The use of nanomaterials to enhance immunotherapeutic efficacy and modulate the tumor immune microenvironment represents a promising strategy. The interaction between cuproptosis and immunotherapy offers a viable approach for improving cancer treatment [64]. Cuproptosis increases tumor cell immunogenicity and activates adaptive immune responses, while also promoting M1 macrophage polarization and enhancing their phagocytic activity. Moreover, by disrupting tumor cell metabolism, cuproptosis induces inflammatory signals that activate the immune system, suppress immunosuppressive cells, and counteract immune evasion mechanisms. Therefore, engineered nanomaterials hold great potential for inducing cuproptosis-immunotherapy synergy. This review summarizes recent advances in nanomedicines designed for cuproptosis-immune combination therapy, including metal oxides, MOFs, elemental metals, LDH, and polymeric nanomaterials. Despite significant progress in preclinical studies, several challenges remain in translating these strategies into clinical practice [102].

Biological safety forms the foundation of nanomaterial development [103]. Copper toxicity is dose-dependent: low intake levels are generally non-toxic, whereas excessive exposure increases serum copper concentrations and induces toxic effects [104]. Therefore, the design of copper-based nanomaterials must prioritize detoxification strategies to meet strict biosafety standards [59]. Three key objectives guide their synthesis: minimizing metal toxicity, optimizing therapeutic dosing, and precisely controlling copper ion release. To reduce metal toxicity, intelligent pre-synthesis toxicity assessments are essential. For example, simultaneous activation of cuproptosis and pyroptosis can trigger inflammatory cytokine storms. To avoid severe complications, real-time monitoring of inflammatory biomarkers after administration allows for timely adjustments in nanomedicine formulation. Surface modifications—such as calcium phosphate coating, polydopamine encapsulation, and ZrO_2_-based barriers—also significantly lower toxicity. Therapeutic dose optimization is closely tied to precise control of copper ion release. Improved tumor targeting and controlled-release systems enable fine-tuned copper delivery, offering dual benefits: reducing off-target effects on copper-dependent enzymes while maximizing therapeutic efficacy [105]. Integrating ^64^Cu-PET imaging with kinetic modeling enables real-time measurement of cytochrome *c* oxidase occupancy, providing feedback for accurate dosage adjustments. Although the Enhanced Permeability and Retention (EPR) effect facilitates tumor accumulation of nanomaterials, several limitations remain. Vascular heterogeneity across patients, tumor types, and even within the same tumor leads to inconsistent EPR performance. This variability also hinders nanoparticle penetration into the tumor core. Moreover, clearance by the mononuclear phagocyte system (MPS) results in unstable tumor accumulation via EPR [106]. To overcome these challenges and achieve precise tumor targeting while minimizing toxicity to healthy tissues, a multi-pronged strategy is required: Firstly, developing multi-stage targeting systems that allow real-time path adaptation, accurate tumor subregion identification, and directed cell targeting [107]. Secondly, implementing personalized precision medicine based on individual patient profiles [108]. This may involve multi-region tumor sampling to detect genetic mutations, identifying high- and low-metabolic zones for targeted dosing, and real-time tracking of cancer stem cell dynamics to develop resistance-resistant therapies [109]. Innovative administration strategies also play a key role in reducing toxicity and improving therapeutic outcomes. For example, altering the injection route can significantly lower metal toxicity [110], with intratumoral delivery showing reduced toxicity compared to intravenous administration [111]. In glioblastoma (GBM), intranasal delivery bypasses the blood-brain barrier (BBB) and leverages organ-specific pathways to counteract immunosuppression in matrix-rich "cold" tumors [112]. For pancreatic cancer, ultrasound-triggered sequential drug release enhances penetration through the stroma and transforms "cold" tumors into "hot" tumors, thereby boosting immune activation [113].

Currently, as the integration of nanotechnology and medicine deepens, the complexity of nanomedical materials is on the rise, making the interactions between nanomaterials and different organs in the human body increasingly challenging to anticipate. Initially, the substantial disparities between experimental animals and humans present various challenges in the clinical translation of nanomedicine-mediated cuproptosis and anti-tumor immune combination therapy. Current in vivo animal models for nanomaterial research predominantly rely on subcutaneous xenograft models. This approach potentially compromises the clinical relevance of experimental results, particularly when delivery strategies depend on passive targeting or the Enhanced Permeability and Retention (EPR) effect. Such preclinical models may lead to significant discrepancies between research findings and predicted clinical performance, as they fail to faithfully recapitulate clinical tumors and their microenvironment. Genetically engineered mouse models (GEMMs) or patient-derived xenografts (PDXs) may serve as effective alternative models to bridge this gap and provide more reliable predictions of nanomaterial performance in future clinical settings. Furthermore, substantial interspecies differences often impede the translational utility of experimental data. To address this challenge, organ-on-a-chip technology can be employed to recreate human hepatobiliary barrier function, thereby reducing prediction errors for copper accumulation-induced liver injury. Additionally, 3D bioprinted tumor models simulating human stromal density and vascular architecture can enhance the accuracy of penetration prediction for copper NPs.

Although combination regimens incorporating cuproptosis-based immunotherapy demonstrate greater anti-tumor efficacy than single-agent approaches, their impact on enhancing tumor immunity remains to be fully elucidated. Most current studies assessing cuproptosis-induced improvements in the tumor immune microenvironment rely on flow cytometry to evaluate short-term changes in immune cell populations. A more detailed investigation is needed to assess long-term immunological effects. First, detailed flow cytometric phenotyping of memory T cells should categorize them into central memory T cells (TCM), effector memory T cells (TEM), and tissue-resident memory T cells (TRM) based on characteristic molecular markers. Furthermore, establishing tumor rechallenge models is critical for effectively evaluating long-term immune efficacy. This process involves eliminating the primary tumor through drug treatment or combined immunotherapy to reach a "medically cured" state. Weeks or months later, the same tumor cells are re-inoculated either contralaterally or at a new anatomical site. A comparative analysis of tumor volume, weight, and other relevant parameters between the initial and rechallenge groups is then conducted. If durable immune protection is present, the rechallenged tumors will show significant growth inhibition or complete rejection. At the same time, T-cell depletion control groups must be included to confirm that the observed protection is T-cell dependent. Moreover, functional validation of memory T cells and investigation into the underlying molecular mechanisms are necessary. This includes measuring cytokines such as IFN-γ, TNF-α, and IL-2; evaluating the cytotoxic activity of T cells isolated from rechallenged animals in co-culture assays; and applying high-throughput sequencing to identify gene expression differences between memory and naïve T cells, thereby revealing key regulatory pathways. In summary, establishing an effective, accurate, and comprehensive immune evaluation framework is essential for assessing long-term anti-tumor immunity and guiding clinical translation.

Furthermore, as a novel cell death mechanism, cuproptosis is intrinsically linked to tumor drug resistancethrough copper transporter-mediated efflux (e.g., CTR1, ATP7A/B expelling cisplatin/oxaliplatin/carboplatin) [114], aberrant expression of cuproptosis regulators (e.g., elevated negative regulators MTF1/Gls/CDKN2A suppressing cell death, or reduced positive regulators FDX1/LIAS/DLAT attenuating induction), and TME heterogeneity (uneven copper distribution, hypovascular copper deficiency, or copper chelators promoting insensitivity). To counter such resistance, multifaceted strategies are essential: combination therapies (e.g., baicalein synergizing with cisplatin to enhance cervical cancer sensitivity), personalized approaches dynamically calibrated via monitoring patient-specific copper homeostasis and gene expression, and novel cuproptosis inducers targeting mitochondrial dysfunction and inhibiting drug efflux pathways. Ultimately, deciphering cuproptosis-resistance mechanisms and designing multimodal anti-tumor agents offer significant potential to maximize therapeutic efficacy while minimizing resistance development.

The current integration of therapeutic strategies centered on cuproptosis shows considerable promise. However, as tumors evolve and their metabolic profiles change, so too does TME. Close monitoring of these TME alterations and the underlying regulatory mechanisms is therefore essential. Understanding these changes may enable cuproptosis to overcome persistent therapy resistance and interrupt tumor progression cycles. Overall, combining nanotechnology with cuproptosis-based immunotherapy represents a meaningful advancement in developing novel anti-tumor therapies. Further elucidation of tumor regulatory mechanisms is critical for achieving precise targeting, improving biosafety, and ensuring clinical relevance [111].

**Table:** Abbreviations

NCT	Nano-catalytic Therapy
SDT	Sonodynamic Therapy
CDT	Chemodynamic Therapy
PDT	Photodynamic Therapy
PTT	Photothermal Therapy
US	Ultrasonography
EPR	Enhanced permeability and retention
ROS	Reactive oxygen species
GPX4	Glutathione peroxidase 4
Cytc	Cytochrome *c*
DAMPs	Damage-Associated Molecular Patterns
GSH	Glutathione
ICD	Immunogenic cell death
TME	The immunosuppressive tumor microenvironment
BBB	Blood-brain barrier
MDSCs	Myeloid-derived suppressor cells
TCA	Tricarboxylic acid cycle
DLAT	Dihydrolipoamide S-acetyltransferase
FDX1	Ferredoxin 1
LIAS	lipoyl synthase
HSPs	Heat shock proteins
PCD	Programmed cell death
mtDNA	Mitochondrial DNA
ATP	Adenosine 5′-triphosphate
CTLs	Cytotoxic T lymphocytes
LDHs	Layered double hydroxides
CRT	Calreticulin
HMGB1	High mobility group box 1 protein
mDCs	Mature dendritic cells
dLNs	Draining lymph nodes

## CRediT authorship contribution statement

**Jiaxin Li:** Writing – original draft. **Xinlong Pang:** Resources. **Zhongwei Xin:** Investigation. **Liang Song:** Formal analysis. **Xiangyan Liu:** Formal analysis. **Xinyu Zhang:** Writing – review & editing. **Zhe Yang:** Writing – review & editing.

## Availability of data and materials

No datasets were generated or analysed during the current study.

## Funding

We acknowledge the funding provided by the 10.13039/501100007129Natural Science Foundation of Shandong (ZR2024QH013) and Incubation Fundation of Shandong Provincial Hospital (2023FY089).

## Declaration of competing interest

The authors declare that they have no known competing financial interests or personal relationships that could have appeared to influence the work reported in this paper.

## Data Availability

The data that has been used is confidential.
